# A Scoping Review of Maternal and Child Health Clinicians Attitudes, Beliefs, Practice, Training and Perceived Self-Competence in Environmental Health

**DOI:** 10.3390/ijerph121215018

**Published:** 2015-12-10

**Authors:** Lamin Daddy Massaquoi, Nancy Christine Edwards

**Affiliations:** School of Nursing, University of Ottawa, ON K1N6N5, Canada; lamin_massaquo@yahoo.com

**Keywords:** environmental health, clinicians, attitude, belief, practice, competence, training

## Abstract

Clinicians regularly assess, diagnose and manage illnesses which are directly or indirectly linked to environmental exposures. Yet, various studies have identified gaps in environmental assessment in routine clinical practice. This review assessed clinicians’ environmental health practices, attitudes and beliefs, and competencies and training. Relevant articles were sought using a systematic search strategy using five databases, grey literature and a hand search. Search strategies and protocols were developed using tailored mesh terms and keywords. 43 out of 11,291 articles were eligible for inclusion. Clinicians’ attitudes and beliefs towards environmental health and routine clinical practice were generally positive, with most clinicians believing that environmental hazards affect human health. However, with the exception of tobacco smoke exposure, environmental health assessment was infrequently part of routine clinical practice. Clinicians’ self-competence in environmental assessment was reported to be inadequate. Major challenges were the time required to complete an assessment, inadequate training and concerns about negative patients’ responses. Clinicians have strong positive attitudes and beliefs about the importance of environmental health assessments. However, more concerted and robust strategies will be needed to support clinicians in assuming their assessment and counselling roles related to a wider range of environmental hazards.

## 1. Introduction

The role of health providers in assessing and mitigating the impact of environmental hazards is controversial. Yet, on a daily basis, clinicians assess, diagnose and manage illnesses, which are directly or indirectly linked to environmental exposures [1]. There are three prominent perspectives regarding how best to prepare clinicians and reorient the health care system towards environmentally-induced diseases. Some argue that an understanding of environmental exposures is fundamental to the training of health professionals, that this constitutes a core competency in primary care clinical practice and can be augmented with support from environmental resource units [2]. Others emphasize the importance of specialty programs, such as occupational health training and the establishment of specialized referral centers [3]. A third perspective is public health-oriented and emphasizes addressing upstream determinants of harmful environmental exposures through strategies such as inter-sectoral policy change [4,5]. 

These perspectives are reflected in a range of documents. Position statements, declarations and related advocacy activities of professional associations address many environmental issues. Internationally, for example, both the World Medical Association and the International Council of Nurses have issued position papers addressing competencies of, and advocacy by health care professionals on environmental issues such as the management of chemicals and health waste, disaster preparedness, tobacco control, safe water, and cancer prevention [6,7]. The Federation of World Public Health Associations issued the Kolkata Call to Action for Healthy Environments (2015), reflecting similar positions taken by member agencies [8]. Environmental core competencies for clinicians are identified in major reports. For instance, The Institute of Medicine (1996) described four categories of key environmental health competencies for clinicians: knowledge and concepts; assessment and referral; advocacy, ethics and risk communication; and knowledge of legislation and regulation [9]. Examples of these competencies are seen in some health professional regulations [10,11]. 

Despite these directions, various authors have identified gaps in environmental assessment practices in routine practice [12]. Although these gaps are a concern for all age groups, they are particularly worrisome for pregnant women, infants and children, given their susceptibility to harmful environmental exposures [13]. Practice gaps are being tackled through the development and dissemination of clinical guidelines [14], the integration of environmental health courses in some training programs [12] and the establishment of environmental health specialist units such as the Pediatric Environmental Health Specialist Units in parts of North America and Europe [3]. Explanations for the insufficient uptake of environmental assessment strategies by clinicians in routine practice include issues of provider payment and assessment time, patient receptivity to this line of questioning, and lack of timely data from environmental hazard surveillance systems that could help pinpoint priority areas of exposure assessment [15,16,17]. 

### Purpose of Review

The overarching aims of our scoping review were to examine:

- The attitudes and beliefs of maternal and child health clinicians in environmental health
- The state of environmental health assessment by these clinicians in their routine practice
- Maternal and child health clinicians’ perspectives about their self-competence in managing environmental hazard(s) associated with health problems
- Maternal and child health clinicians’ challenges to the inclusion of environmental health in routine clinical practice.

We began by searching for existing reviews on this topic. We located one systematic review on environmental health practices, training, attitudes, beliefs and competencies of clinicians [18]. This article also included primary data from a cross-sectional study. These authors reported gaps in child healthcare providers’ self-efficacy and knowledge about environmental hazards. Our review considerably extends the review by Trasande *et al.* [18] by increasing the clinician categories included, the geographic scope, the number of databases searched, and the search strategies employed. Trasande *et al.* [18] focused on child health physician specialists in the United States. Only one of their eight articles included other types of clinicians (family physicians, naturopaths and nurses). We included all maternal and child healthcare clinicians (family physicians, general physicians, pediatricians, obstetricians, gynaecologists, nurses and midwives); and all countries. Also, instead of searching just one database and using three search terms, as was done in the previous review, we included five databases and a more comprehensive search strategy, which included hand and grey literature searches. 

## 2. Methods

### 2.1. Search Strategies

Search strategies and protocols were developed in consultation with an expert librarian in Health Sciences. Mesh terms and keywords were tailored to each database (see Supplementary). 

Five databases were searched: PUBMED, MEDLINE, SCOPUS, CINAHL and EMBASE. To search for reports and surveys not published in the five databases, grey literature and hand search were conducted using three electronic search engines (Google, Yahoo and Microsoft) and a selection of professional associations. In addition, references of selected articles were reviewed for relevant articles that could have been missed during database searches.

Duplicate articles were removed and titles and abstracts were screened for relevance based on eligibility criteria. In the few instances when we could not determine eligibility from abstracts, we looked at full texts. 

### 2.2. Study Selection

Eligibility criteria used to select articles for review and extraction are listed below:

Inclusion Criteria:

- English articles published between 2000 and 2014.
- Any geographic location.
- Empirical study conducted on trained and practicing clinicians including general, family and primary care physicians, obstetricians, pediatricians, midwives and nurses.
- Clinicians must be primarily attending to clients in a clinical setting.
- Studies assessing competence, attitude, belief, opinions, practice, education and/or training in environmental health and/or in environmental exposures.
- Examines at least one environmental health hazard exposure.

Exclusion Criteria:

- Studies that only describe clinicians’ non-clinical practice personal behavior to reduce risk of environmental exposure (e.g., personal smoking behavior).
- Opinions, commentaries, editorials, viewpoints, letters and notes.
- Studies describing only specialist occupational health clinicians and/or public health workers whose practice is not in a clinical setting.

### 2.3. Data Extraction, Collation and Reporting

Data from eligible studies were extracted by one of the authors (LM) using an extraction form developed by both authors. Extracted data for a subset of articles were verified for accuracy and completeness by the other author (NE). Extraction forms included information on authorship and study objectives; methods (study design, sample size, response rate); study population, location, and type(s) of environmental hazard; clinician type and practice setting; and clinicians’ attitudes and beliefs, practices, competencies and challenges. Extraction of data for clinician environmental health attitudes/beliefs, practices, competencies and challenges were achieved by searching articles for answers to specific statements developed both a priori and during reading of articles. Statements used for data extraction are described in Box 1. 

Quantitative studies used one of two measurements: percentages and/or Likert scales ranging from 1 to 5 or from 1 to 10. Extracted data were reported as “most” or “majority”, if proportions were greater than 50%, greater than 2.5 on a Likert scale of 5, or greater than 5 on a Likert scale of 10.

Box 1 **Categories used for data extraction.**

**Attitudes and beliefs of clinicians**
Environmental hazard(s) exposure(s) affect human healthEnvironmental health history taking should be part of routine practiceCounselling clients on hazard exposures**Clinician practice patterns**
Environmental history taking in routine practiceEnvironmental exposure counselling in routine practiceReferral to specialist centers/units/personnel**Clinician self-rated competence**
Knowledge of environmental hazardsAbility to take an environmental health history**Clinician training**
Undergraduate or specialist training in environmental health Clinicians’ interest in more environmental health trainingSource(s) of continuing environmental health education**Challenges towards the inclusion of environmental health assessment in routine clinical practice**

## 3. Results

**Figure 1:**
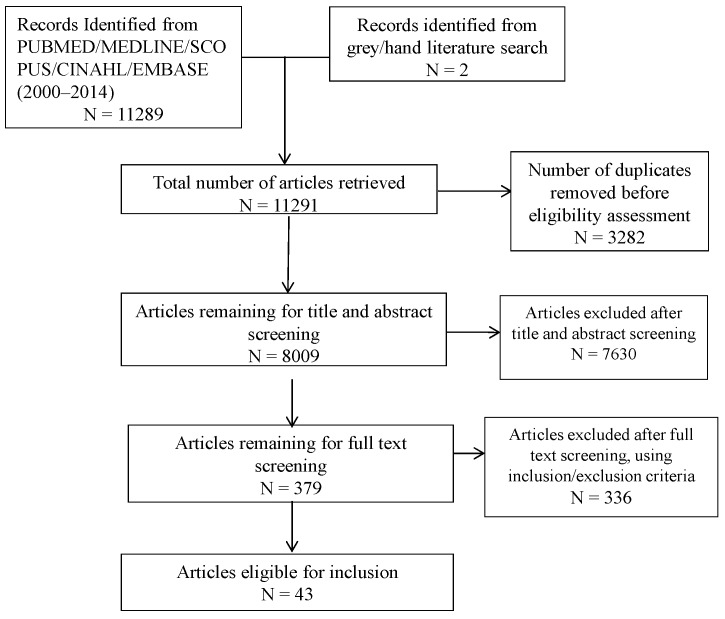
Study selection flowchart.

From a total of 11,291 articles, 43 articles were eligible for inclusion, see Figure 1. Among these, 41 studies used quantitative data collection and 2 studies employed a mixed methods data collection. Among the 41 quantitative studies, thirty nine were cross-sectional. There were three intervention studies, one used cross-sectional data and two were before and after studies. Twenty-nine studies included single provider groups: nurses (N = 6), pediatricians (N = 12); midwives (N = 2); obstetricians (N = 1); and primary, general or family physicians (N = 8). Fourteen articles included more than one type of clinician (see Table 1, Supplementary Table 1 (ST1)). Most studies recruited from a population of all clinician members of professional associations. Response rates ranged from 11% to 100% with an average of 58%. A few authors utilized convenience sampling techniques. There was no consistent pattern of differences in characteristics among clinician categories within or between articles. As such, results are combined for all clinician groups. 

The majority (60%) of articles were from North America (24 articles from the United States of America (U.S.A) and two articles from Canada). Seven articles were from Europe, seven from Asia and the Middle East, two from Africa and one from South America (Argentina) (Table 1, Table S1). No study was conducted in multiple countries.

**Table 1:** Showing study characteristics and type(s) of clinicians studied.

Study Author (s)	Author(s) Number	Study Location	Pediatrician	Nurses	More than 1 Type of Clinician	Others
[19]	1	U. S. A			√	
[20]	2	Tanzania			√	
[21]	3	U. S. A			√	
[22]	4	U. S. A			√	
[23]	5	Thailand				√ ^a^
[24]	6	U. S. A		√		
[25]	7	U. S. A	√			
[26]	8	U. S. A	√			
[27]	9	Germany	√			
[28]	10	Bangladesh			√	
[29]	11	U. S. A	√			
[30]	12	U. K			√	
[31]	13	U. S. A				√ ^a^
[32]	14	U. S. A				√ ^a^
[33]	15	Germany				√ ^b^
[34]	16	U. S. A			√	
[35]	17	Canada			√	
[36]	18	U. S. A	√			
[37]	19	U. S. A			√	
[38]	20	Italy				√ ^a^
[39]	21	U. S. A	√			
[40]	22	U. S. A	√			
[41]	23	U. S. A				√ ^b^
[42]	24	U. S. A	√			
[43]	25	U. S. A			√	
[44]	26	Canada		√		
[45]	27	New Zealand			√	
[46]	28	Pakistan				√ ^a^
[47]	29	U. S. A	√			
[48]	30	Sweden		√		
[49]	31	U. S. A		√		
[50]	32	Bahrain				√ ^a^
[51]	33	Germany				√ ^a^
[52]	34	Argentina			√	
[17]	35	U. A. E		√		
[18]	36	U. S. A			√	
[53]	37	Egypt	√			
[54]	38	China				√ ^a^
[55]	39	U. S. A			√	
[56]	40	U. S. A	√			
[15]	41	U. S. A		√		
[16]	42	U. S. A				√ ^c^
[57]	43	China	√			
Total			12	6	14	11

Notes: ^a^ General/Family Physicians; ^b^ Midwives; ^c^ Obstetricians.

Environmental health hazards studied included: tobacco smoke; metals and organic toxins; indoor and outdoor air, water, and soil pollutants; sun radiation; domestic and occupational environmental hazards; and radioactive chemicals. Tobacco smoke exposure was the most studied hazard with 30 (70%) articles examining tobacco smoke exposure (Table 2). Seventeen of the 30 articles focused exclusively on tobacco smoke exposure and 13 studies included other environmental health hazards: heavy metals, organic pollutants, radiation, allergens, carbon monoxide, and poor water and/or air quality. Four studies that examined other environmental health hazards did not include tobacco smoke exposure and six articles examined a single non-tobacco exposure: arsenic, occupational hazards, sun radiation, air pollution, radon, and electromagnetic radiation. Three other studies reported exclusively on pesticide exposure (Table S2). 

**Table 2:** Type of environmental hazard assessed in studies.

Author #	Type of Environmental Hazard Assessed					
	Tobacco	Pesticides	Lead	Mold	Other Metal(s)	Other Hazard(s)
1		√			√	√
2		√ ^a^				
3	√		√			√
4	√ ^a^					
5						√
6						√
7	√		√	√		√
8	√ ^a^					
9	√ ^a^					
10					√ ^a^	
11						√ ^a^
12						√ ^a^
13	√	√	√		√	√
14	√ ^a^					
15	√ ^a^					
16		√ ^a^				
17	√	√		√	√	√
18					√	√
19		√ ^a^				
20	√				√	√
21		√	√	√		
22	√	√	√	√	√	√
23	√ ^a^					
24	√ ^a^					
25	√ ^a^					
26	√		√	√	√	√
27	√ ^a^					
28					√ ^a^	
29	√		√			√
30	√ ^a^					
31	√ ^a^					
32	√ ^a^					
33						√ ^a^
34	√ ^a^					
35	√ ^a^					
36	√	√	√	√	√	√
37	√	√		√		√
38	√ ^a^					
39	√ ^a^					
40	√			√		√
41	√ ^a^					
42	√	√	√	√	√	√
43	√	√	√	√	√	√
Total # of Studies # of studies on single hazard	30 17	12 3	10 0	10 0	12 2	20 3

Note: ^a^ Study assessed only a single exposure.

Little or no psychometric data was provided for study instruments. A number of questionnaire items were similar across some studies. These included items on attitudes and beliefs, practice and self-rated competence especially for studies assessing overall environmental hazard competence of clinicians. 

### 3.1. Clinicians’ Attitudes and Beliefs

We extracted information about clinicians’ beliefs about environmental hazards and their effects on health, their attitudes regarding the inclusion of environmental history taking in routine clinical practice and whether they considered counselling about reducing environmental hazards to be important.

Twenty-five articles reported clinicians’ environmental health beliefs and attitudes. Twenty (80%) of these articles reported clinicians’ beliefs about the role of environmental hazards and their effects on human health. In all but one of these 20 articles, the majority of clinicians believed that environmental hazard exposures affected human health (Table 3, Table S3). Thirteen out of the 20 articles examined more than one environmental hazard, three studies examined only tobacco smoke exposure and four studies examined other singular exposures.

Eleven of the 25 (52%) studies reported clinicians’ attitudes towards the inclusion of environmental history taking in routine practice. All of these articles examined more than one environmental health hazard. Most (9/11, 81.8%) reported that a high proportion of clinician respondents believed environmental exposure history taking is important and should be part of routine practice. 

Similarly, in another subset of 15 studies, most clinicians (12/15, 80%) believed that providing environmental hazard counselling could help reduce patient exposures. Six of these 15 studies examined only tobacco smoke exposure, one study was on sun radiation and eight other studies examined more than one environmental hazard (Table 3).

### 3.2. Practices

Environmental health clinical practice patterns assessed included routine environmental history taking, counselling on environmental hazard exposure and patient referral practices (Table 3, ST3). Thirty articles reported environmental health practices. Twenty-four of the 30 articles (80%) reported environmental health history taking in routine practice (Table 3). Of the 24 articles, 12 were exclusively on tobacco smoke exposure, two were on pesticide exposures and 10 examined more than one environmental health hazard. Eight of the 12 articles (66.6%) examining only tobacco smoke exposures, reported that a majority of clinicians routinely took environmental health histories. Similarly, six of the 10 studies (62.5%) examining more than one hazard reported that most clinicians routinely took an environmental history, but mostly for known hazards such as tobacco smoke, lead and mold. Both of the studies on pesticide exposures reported a low proportion of clinicians taking routine environmental health history. 

Twenty-one articles reported counselling practices. Fourteen articles reported exclusively on tobacco with ten of these studies (71.4%) indicating that most clinicians provided routine counselling on tobacco smoke exposure (Table 3). Two of the 21 articles, focused on other exposures excluding tobacco: neither of these two articles reported a majority of clinicians providing routine counselling. Five other studies examined more than one environmental hazard- with four of these reporting that most clinicians provided routine counselling; tobacco smoke was the most common hazard for which clinicians provided routine counselling.

Only eight out of the 30 studies on practice patterns (26.6%) had information on clinician referral practice for patients with environmental hazard exposures. Five of these studies assessed more than one hazard and in all cases, a majority of clinicians indicated they would refer to environmental specialists’ if they were available. Three of the eight studies were exclusively on tobacco; a low proportion of clinicians in these studies reported intentions to refer.

### 3.3. Self-Assessed Competencies and Training

Clinicians’ self-rated competencies in managing clients’ environmental health related problems were reported to be inadequate in the majority of studies (13/21, 61.9%). All but one of the eight exceptions included tobacco smoke. 

Twenty-one articles reported on prior environmental training: eight of these focused on tobacco smoke exposure, four on other singular exposures and nine examined more than one hazard. A similar pattern was observed regardless of hazard assessed. In the majority of these studies (62%), most clinicians reported inadequate training in environmental health but among those asked, most indicated they were interested in more training (Table 3, Table S3). 

Ten articles reported on the most helpful sources for continuing educational resources in environmental health. The most helpful resources identified were practice guidelines and scientific articles accessible on the internet. Short term training such as workshops, conferences and seminars were preferred to long term training. Clinicians also relied on expert colleagues in environmental health. 

### 3.4. Challenges to Practice

Twenty-one articles (10 on tobacco, 8 on tobacco in combination with other exposures, and 3 on other exposures) reported challenges. All identified lack of time and/or inadequate training as a challenge (Table S4). In addition, most articles and especially those on tobacco smoking exposure indicated negative responses from patients as a challenge to counselling on hazard exposure reduction. Other challenges such as inadequate environmental resources (lab testing facilities, equipment, infrastructure, *etc.*), and a lack of reimbursement and consultation services were more commonly reported in articles focused on more than one type of environmental hazard. 

**Table 3:** Number of articles assessing hazard(s) and clinician responses to questionnaire items.

Categories	# of Articles	Hazard Exposure		
		Tobacco Smoke # of Articles ^a^	Tobacco Smoke + Other Exposures ^a^	Other Exposures (Not Including Tobacco Smoke) ^a^
**Attitudes and Beliefs**	**25**			
Environmental exposure(s) affect(s) human health	20	3 (3)	9 (9)	8 (7)
Environmental health history taking should be part of routine practice	11	0 (0)	8 (8)	3 (1)
Counselling of patients on Environmental exposures can help reduce exposures	15	6 (5)	6 (4)	3 (3)
**Practices**	**30**			
Clinician takes routine environmental exposure history	24	12 (8)	9 (6)	3 (0)
Includes environmental exposure counselling in routine practice	21	14 (10)	5 (4)	2 (0)
Refers/would refer cases associated with environmental exposures to specialists	8	3 (0)	4 (4)	1 (1)
**Competence/Training**	**35**			
Sufficiently informed on environmental exposures	21	6 (2)	9 (5)	6 (1)
Prior training in environmental health history taking	21	8 (2)	6 (0)	7 (1)
Requires/interested in more training	16	6 (6)	4 (4)	6 (5)

Note: ^a^ Number in brackets indicate number of studies with high proportion of clinicians agreeing to item category.

## 4. Discussion

This review assessed clinicians’ environmental health practices, attitudes and beliefs, and competencies and training. The inclusion of environmental health in routine practice as well as the reported competence of clinicians was similar for all clinician groups. However, there were considerably more studies involving physicians than other health providers, so this comparison is tentative. 

Many studies addressed only a single environmental exposure (notably tobacco). Among the few articles examining more than one exposure, the number assessed was small. This reflects a dilemma; which hazards should be prioritized for study and for clinical practice improvements. The predominance of articles on tobacco was not surprising given its prevalence, known disease consequences, and strong public health efforts to reduce both primary and secondary tobacco smoke exposure [58]. Those hazards for which assessment and counseling practices were more commonly reported included tobacco, lead, pesticide use and sun exposure. Clinicians may think they have a wider set of concrete mitigation strategies to recommend to patients for these environmental hazards in contrast to others for which individual patients have no direct control (e.g., outdoor air pollution). 

Findings indicate the importance of assessing not only attitudes but also actual practices. We do not know the extent to which clinicians may over or under-report their actual assessment and counseling practices or how a reporting bias may be influenced by knowledge of guidelines or attendance at continuing education programs. 

Preferred sources of environmental health continuing education for most clinicians were similar to those commonly suggested in studies of professional development [59,60]. These included short term training programs, scientific texts retrieved from the internet, and guidelines from professional associations. Since standards, regulatory mechanisms, and the nature of environmental hazards are changing regularly, better and novel communication and data sharing mechanisms are needed. Given the lack of content in undergraduate programs on environmental issues [12], clinicians are starting out to practice in a knowledge deficit position.

### 4.1. Study Limitations

We included only English language articles published from January 2000 to November of 2014. We combined clinicians in the early analysis of our results. We were not able to discern differences across clinician categories but our categorization of responses into “most” or “few” using a 50% cut-point, may have masked differences. We excluded articles that focused on specialist occupational health clinicians. We may have seen a different pattern of responses in this sub-group. However, they were deliberately excluded given our focus on clinicians regularly caring for pregnant women, infants and children.

While there were some commonly asked questions about environmental assessment and management across studies, the use of standard, validated tools was not apparent. This makes comparisons across studies more tentative. However, some of the differences in patterns of responses (e.g., responses to questions about tobacco *versus* other hazards) were so pronounced that more refined questionnaires are unlikely to have led to different conclusions. 

### 4.2. Future Studies

Clinicians face pragmatic realities when they try to include environmental health assessment and counselling in routine clinical practice. Numerous studies in the field of tobacco control have addressed some of these challenges such as those examining brief motivational counselling [61] or the inclusion of tobacco assessment criteria in standard prenatal records [62,63]. Similar efforts are needed in relation to other environmental hazards. 

Clinical practice guidelines are one means of informing practice standards and expectations [64]. While stand-alone guidelines related to environmental hazards may be useful, the integration of environmental assessment and counselling practices within other guidelines should be considered and needs to be tested. Furthermore, given new etiological research on environmental hazards and surveillance data that helps track exposures [65], studies are needed on the effective use of environmental health information by clinicians. These would improve environmental health data communication to clinicians. 

We found few comparative studies. Multi-jurisdictional studies are needed to examine the impact of differing environmental regulations on clinicians practice, both within and between clinician types. There were very few studies reported from low income and middle countries. Given health human resource challenges and weaker enforcement of environmental controls, studies in these settings should be a priority. 

## 5. Conclusions

Clinicians seem to have strong positive attitudes and beliefs about the importance of environmental health assessments. Yet, with the exception of tobacco smoke exposure, risk assessment and counselling for other environmental exposures were infrequently part of routine clinical practice. Achieving practice changes in relation to tobacco smoke has required regulations, public health campaigns and public pressure. We must ask ourselves if the period it has taken us to achieve these practice changes is an adequate health system and clinician response to a known carcinogen. Etiological studies will continue to deepen our understanding of causal relationships between environmental exposures and disease outcomes and new hazards will be identified as we have seen more recently with Bisphenol A, and flame retardants [66,67]. More concerted, robust and timely strategies will be needed to support clinicians in assuming their assessment and counselling roles related to newly confirmed hazards. The health stakes are too high for environmental hazard assessment and counselling to be discretionary practices. 

## Supplementary Files

Supplementary File 1
