# Supplementary material for: A Scoping Review of Maternal and Child Health Clinicians Attitudes, Beliefs, Practice, Training and Perceived Self-Competence in Environmental Health
Source: Int J Environ Res Public Health. 2015 Dec 10;12(12):15769–81. doi: 10.3390/ijerph121215018 (PMC4690954; doi:10.3390/ijerph121215018)
Supplement: Supplementary File 1 [file ijerph-12-15018-s001.pdf]

Supplementary Information

A Scoping Review of Maternal and Child Health Clinicians Attitudes, Beliefs, Practice, Training and Perceived Self-Competence in Environmental Health

CINAHL

Search ID	Search Item	Limiters	Retrieved Items
S1	(MH "Midwifery+")		3080
S2	(MH "Midwives+")		8293
S3	(MH "Novice Clinicians+") OR (MH "Expert Clinicians+")		13815
S4	(MH "Nurses+")		150,591
S5	(MH "Physicians+")		52,175
S6	(MH "Pediatricians")		1334
S7	(MH "Pediatrics+")		7459
S8	(MH "Medicine+") OR (MH "Obstetrics") (MH "Gynecology")		82,879
S9	(MH "Knowledge+")		30426
S10	(MH "Education+")		468,242
S11	(MH "Attitude+")		209,479
S12	(MH "Behavior+")		453,176
S13	(MH "Professional Practice+") OR (MH "Nursing Practice") OR (MH "Medical Practice") OR (MH " Practice Patterns") OR (MH "Professional Practice, evidence-Based") OR (MH "Professional Practice, theory-Based") OR (MH "Scope of Practice+")		146,321
S14	(MH "Environmental Health)		3718
S15	(MH "Environmental Exposure)		22,564
S16	(MH "Environmental Pollution")		48,441
S17	(MH "Environmental Pollutants, Pesticides (Non-Cinahl)+") OR (MH "Environmental Pollutants+")		11955
S18	S1 OR S2 OR S3 OR S4 OR S5 OR S6 OR S7 OR S8		288,338
S19	S9 OR S10 OR S11 OR S12 OR S13		891,869
S20	S14 OR S15 OR S16 OR S17		57,293
S21	S18 AND S19 AND S20		1224
S22	S18 AND S19 AND S20	20000101–20141231	1042

EMBASE

Search ID	Search Item	Retrieved Items
1.	Obstetrician.mp. or exp obstetrics or exp obstetrician/	38,263
2	Gynaecologist.mp or gynecologist	3697
3	Gynaecology.mp or exp gynecology	40,926
4	Pediatrician.mp. or exp pediatrician	17,142

EMBASE *Cont.*

Search ID	Search Item	Retrieved Items
5	Pediatrics.mp. or exp pediatrics/	117,181
6	Exp physician or physician.mp.	597,061
7	Midwife.mp. or exp midwife/	24,759
8	Midwifery.mp	7195
9	Exp nurse or nurse.mp.	226,203
10	Nursing or nursing.mp	512,727
11	Environmental health.mp. or exp environmental health/	39,082
12	Environmental exposure.mp or exp environmental exposure/	79,988
13	Environmental pollution. mp. Or exp pollution/	285,689
14	Exp pollution or pollution.mp.	309,506
15	Pollutants.mp or exp pollutant	257,037
16	Exp knowledge or knowledge.mp.	536,070
17	Training.mp. training	393,203
18	Behaviour.mp. or behavior	315,360
19	Exp good clinical practice or exp nursing practice or exp health care practice or exp practice guideline or exp professional practice or exp clinical practice or exp advanced practice nursing or practice.mp. or exp medical practice or exp general practice	1,132,757
20	Esp education or education.mp.	1,193,419
21	Exp competence/ or exp professional competence/ or exp clinical competence/ or exp nursing competence or competence.mp	124,361
22	Skill.mp or skill	76928
23	16 or 17 or 18 or 19 or 20 or 21 or 22	3,016,509
24	11 or 12 or 13 or 14 or 15	509,114
25	1 or 2 or 3 or 4 or 5 or 6 or 7 or 8 or 9 or 10	1,325,433
26	23 and 24 and 25	2745
27	Limit 26 to (English language and year = “2000–Current”)	1946

MEDLINE

Search ID	Search Item	Retrieved Items
1	Exp obstetrics or exp delivery, Obstetrics or obstetrician.mp.	80,229
2	Exp Gynecology or gynaecologist.mp.	14,515
3	Gynecology or gynecologist.mp.	15,591
4	Exp pediatrics or pediatrician.mp.	48,024
5	Physician.mp. or exp Physicians	270,577
6	Midwifery.mp or exp midwifery	18,615
7	Midwife.mp.	4046
8	Nurse.mp. or exp nurses/	163,491
9	Exp Nursing or Nursing.mp.	474,488
10	Environmental health.mp. exp environmental health	24,718
11	Environmental exposure.mp. or exp Environmental exposure	180,839
12	Environmental pollutants.mp. or exp Environmental pollutants	192,446
13	Pollutants	173,019
14	Pollution	100,382
15	Knowledge.mp. or knowledge	474,020

MEDLINE *Cont.*

Search ID	Search Item	Retrieved Items
16	Training.mp.	285,624
17	Behaviours.mp	26,693
18	Practice.mp.	706,875
19	Exp attitude or attitude.mp.	294,943
20	Belief.mp. or exp culture	138,345
21	Education.mp. or exp education	812,543
22	Competence.mp.	119,043
23	Competency.mp. or exp competency-based education/	23,641
24	Skills.mp.	113,012
25	15 or 16 or 17 or 18 or 19 or 20 or 21 or 22 or 23 or 24	2,140,176
26	10 or 11 or 12 or 13 or 14	909,407
27	1 or 2 or 3 or 4 or 5 or 6 or 7 or 8 or 9	380,829
28	25 and 26 and 27	1927
29	Limit 28 to year = “2000–Current”	1263

Scopus

((TITLE-ABS-KEY (obstetrician*) OR TITLE-ABS-KEY (pediatrician*) OR TITLE-ABS-KEY (physician*) OR TITLE-ABS-KEY (paeditrician*) OR TITLE-ABS-KEY (midwife*) OR TITLE-ABS-KEY (nurs*) OR TITLE-ABS-KEY (gynaecolog*) OR TITLE-ABS-KEY (clinician*) OR TITLE-ABS-KEY (gynaecolog*))) AND ((TITLE-ABS-KEY (knowledge*) OR TITLE-ABS-KEY (training*) OR TITLE-ABS-KEY (attitude*) OR TITLE-ABS-KEY (practic*) OR TITLE-ABS-KEY (educat*) OR TITLE-ABS-KEY (competenc*) OR TITLE-ABS-KEY (belief*) OR TITLE-ABS-KEY (skill*))) AND ((TITLE-ABS-KEY (environmental health*) OR TITLE-ABS-KEY (environmental exposure) OR TITLE-ABS-KEY (environmental pollut*) OR TITLE-ABS-KEY (pollut*))) AND (LIMIT-TO (PUBYEAR , 2014) OR LIMIT-TO (PUBYEAR , 2013) OR LIMIT-TO (PUBYEAR , 2012) OR LIMIT-TO (PUBYEAR , 2011) OR LIMIT-TO (PUBYEAR , 2010) OR LIMIT-TO (PUBYEAR , 2009) OR LIMIT-TO (PUBYEAR , 2008) OR LIMIT-TO (PUBYEAR , 2007) OR LIMIT-TO (PUBYEAR , 2006) OR LIMIT-TO (PUBYEAR , 2005) OR LIMIT-TO (PUBYEAR , 2004) OR LIMIT-TO (PUBYEAR , 2003) OR LIMIT-TO (PUBYEAR , 2002) OR LIMIT-TO (PUBYEAR , 2001) OR LIMIT-TO (PUBYEAR , 2000)) AND (LIMIT-TO (LANGUAGE , "English"))

Total articles retrieved = 4626

Pubmed

Search: **(((((((((clinician*[Text Word]) OR ((("gynecology"[MeSH Terms]) OR gynecology*[Text Word]) OR gynaecology[Text Word])) OR (("obstetrics"[MeSH Terms]) OR obstetric*[Text Word])) OR (((("nurses"[MeSH Terms]) OR nurses*[Text Word]) OR "nursing"[MeSH Terms]) OR nursing*[Text Word])) OR ((((midwife[MeSH Terms]) OR midwifery[MeSH Terms]) OR midwife[Text Word]) OR midwifery[Text Word])) OR (("physicians"[MeSH Terms]) OR physician*[Text Word])) OR (("pediatrics"[MeSH Terms]) OR pediatric*[Text Word])) OR paediatric*[Text Word])) AND (((((((("environmental health"[MeSH Terms]) OR "environmental pollution"[MeSH Terms]) OR environmental exposure[MeSH Terms]) OR "environmental pollutants"[MeSH Terms]) OR environmental health*[Text Word]) OR environmental exposure*[Text Word]) OR environmental pollution*[Text Word]) OR environmental pollutants*[Text Word]) OR pollution*[Text Word]) OR pollutants*[Text Word])) AND ((((((((((("knowledge"[MeSH Terms]) OR knowledge*[Text Word]) OR "attitude"[MeSH Terms]) OR attitude*[Text Word]) OR professional practice[MeSH Terms]) OR practice*[Text**

Word]) OR "culture"[MeSH Terms]) OR belief*[Text Word]) OR "clinical competence"[MeSH Terms]) OR competenc*[Text Word]) OR skill*[Text Word]) OR "education"[MeSH Terms]) OR educat*[Text Word]) Filters: **Publication date from 2000/01/01 to 2014/12/31; English**

Total articles retrieved = 2412

Table S1. Showing Author, study method, sample population and study location.

Author Name	Author #	Study Method	Sample Population	Location
(Chai *et al.* 2001)	1	Cross-sectional-Quantitative	General practitioners, Pediatricians, Physicians, Nurses	Northwest—U.S.A
(Ngowi *et al.* 2001)	2	Cross-sectional-Quantitative	Physicians, Nurses, Midwives	Tanzania
(Woolf and Cimino 2001)	3	Cross-sectional-Quantitative	Pediatricians, Nurses	Boston—U.S.A
(Perez-Stable *et al.* 2001)	4	Cross-sectional-Quantitative	Physicians, Pediatricians	California—U.S.A
(Aekplakorn *et al.* 2002)	5	Cross-sectional-Quantitative	General Physicians	Thailand
(Van Dongen 2002)	6	Cross-sectional-Quantitative	Nurses	U.S.A
(Kilpatrick *et al.* 2002)	7	Cross-sectional-Quantitative	Pediatricians	Georgia—U.S.A
(Cabana *et al.* 2004)	8	Cross-sectional-Quantitative	Pediatricians	U.S.A
(Hannöver *et al.* 2004)	9	Cross-sectional-Quantitative	Pediatricians	Germany
(Murshed *et al.* 2004)	10	Cross-sectional-Quantitative	Clinicians	Dhaka—Bangladesh
(Balk *et al.* 2004)	11	Cross-sectional-Quantitative	Pediatricians	U.S.A
(Stevens *et al.* 2004)	12	Cross-sectional-Quantitative	General Practitioners, Nurses	U.K
(Hamilton *et al.* 2005)	13	Cross-sectional-Quantitative	Physicians	Texas—U.S.A
(Soto Mas *et al.* 2005)	14	Cross-sectional-Quantitative	Physicians	New Mexico—U.S.A
(Thyrian *et al.* 2006)	15	Cross-sectional-Quantitative	Midwives	Pomerania—Germany
(Balbus *et al.* 2006)	16	Cross-sectional-Mixed	Physicians, Physician Assistants, Pediatricians, Nurses	Washington—U.S.A
(Buka *et al.* 2006)	17	Cross-sectional-Quantitative	Nurses, Physicians	Alberta—Canada
(Hu *et al.* 2006)	18	Cross-sectional-Quantitative	Pediatricians	New York—U.S.A
(Karr *et al.* 2006)	19	Cross-sectional-Quantitative	Physicians, Nurses	Northwest—USA
(Nicotera *et al.* 2006)	20	Cross-sectional-Quantitative	Physicians	Calabria—Italy
(Trasande *et al.* 2006b)	21	Cross-sectional-Quantitative	Pediatricians	Wisconsin—U.S.A
(Trasande *et al.* 2006a)	22	Cross-sectional-Quantitative	Pediatricians	New York—U.S.A
(Abatemarco *et al.* 2007)	23	Cross-sectional-Quantitative	Midwives	New Jersey—U.S.A
(Collins *et al.* 2007)	24	Cross-sectional-Quantitative	Pediatricians	Philadelphia—U.S.A
(Garg *et al.* 2007)	25	Cross-sectional-(Intervention)-Quantitative	Pediatricians, Physicians	Pennsylvania—U.S.A

Table S1. *Cont.*

Author Name	Author #	Study Method	Sample Population	Location
(Canadian Nurses Association 2008)	26	Cross-sectional-Quantitative	Nurses	Canada
(Glover *et al.* 2008)	27	Cross-sectional-Quantitative	General Practitioners, Midwives	New Zealand
(Rafique *et al.* 2008)	28	Cross-sectional-Quantitative	Physicians	Pakistan
(Trasande *et al.* 2008)	29	Cross-sectional-Quantitative	Pediatrician	Minnesota—U.S.A
(Carlsson *et al.* 2010)	30	Cross-sectional-Quantitative	Nurses	Sweden
(Deckter *et al.* 2009)	31	Cross-sectional-Quantitative	Nurses	Cincinnati—U.S.A
(Fadhil 2009)	32	Cross-sectional-Quantitative	Physicians	Bahrain
(Kowall *et al.* 2010)	33	Cross-sectional-Quantitative	General-Practitioner	Germany
(Mejia *et al.* 2010)	34	Cross-sectional-Quantitative	Gynecologists, Obstetricians, Residents, Non-specific	Argentina
(Sreedharan *et al.* 2010)	35	Cross-sectional-Quantitative	Nurses	U.A.E
(Trasande *et al.* 2010)	36	Cross-sectional-Systematic review	Pediatricians, General practitioners	Michigan—U.S.A
(Abbas and Alghobashy 2012)	37	Cross-sectional-(Before and After) Quantitative	Pediatrician	Zagazig—Egypt
(Huang *et al.* 2013)	38	Cross-sectional-Quantitative	Physicians	Qingdao—China
(Kruger *et al.* 2012)	39	Cross-sectional-Quantitative	Obstetricians, Gynecologists, Pediatricians, General Practitioners	U.S.A
(Roberts *et al.* 2013)	40	Cross-sectional (Before and After) Quantitative	Pediatricians	U.S.A
(Blaine *et al.* 2014)	41	Cross-sectional-Quantitative	Nurses	U.S.A
(Stotland *et al.* 2014)	42	Cross-sectional-Mixed	Obstetricians	California—USA
(Trasande *et al.* 2014)	43	Cross-sectional-Quantitative	Pediatricians	Northwest—China

Table S2. Showing type of exposures assessed and type of exposures most/least discussed by clinicians with their clients.

Author #	Exposures Assessed	Exposures Most Discussed by Clinicians	Exposures Least Discussed by Clinicians
1	Allergens, carcinogens, Electromagnetic fields, food, hormones, industrial waste, metals, PCBs, pesticides, radiation, solvents, water	drinking water, allergens, food, industrial waste, pesticides	hormones, PCPs, EM, radiation
2	Pesticides	n/a	n/a
3	Lead, tobacco smoke, occupation, radon, car seat use, toxins, toxins, allergens, household exposures	Tobacco Smoke, lead poisoning, occupation, car seat use	hobbies, radon
4	Tobacco Smoke	n/a	n/a
5	Occupational exposures	n/a	n/a
6	Environment & health	n/a	n/a
7	Tobacco Smoke, pets, water, lead, housing, sun, television, occupation, molds, heat, air pollution, hobbies, carbon monoxide	Tobacco Smoke, water, lead, housing, molds, heat, air pollution	carbon monoxide, hobbies, outdoor air, sun, nitrates, radiation, asbestos, PCBs, HG, pesticides
8	Tobacco Smoke	n/a	n/a
9	Tobacco Smoke		
10	Arsenic	n/a	n/a
11	Sun Exposure	Smoking	Sun protection
12	Air pollution	n/a	n/a
13	Tobacco Smoke, occupation, nutrition, sun, pets, outdoor pollution, hobbies, housing, pesticides, lead, water, radiation, asbestos, fetal exposures, herbicides, heat source, indoor pollution, emissions, mercury, nitrates, agent orange, Arsenic	Tobacco Smoke, nutrition, occupation	housing, hobbies, fetal exposures, drinking water,
14	Tobacco Smoke	n/a	n/a
15	Tobacco Smoke	n/a	n/a
16	Pesticides	n/a	n/a
17	Indoor air pollution, outdoor air pollution, allergens (pollen, dust, molds), tobacco smoke, medications, pesticides, mercury, other metals, dioxins, bacteria, electronics, sunlight, household exposures, antibiotics, hormones	Tobacco Smoke, air pollution, allergens, pesticides, industrial waste	radiation from electronics, GM foods
18	Bioterrorism, air quality, environmental toxins, anthrax, nuclear event, potassium iodide	n/a	n/a
19	Pesticides	n/a	n/a

Table S2. *Cont.*

Author #	Exposures Assessed	Exposures Most Discussed by Clinicians	Exposures Least Discussed by Clinicians
20	Noise, passive smoking, air pollution, benzene,; radon; ELF; EMF	occupational exposures, indoor and outdoor air pollution, tobacco Smoke, allergens	radon, domestic heating
21	Pesticides, lead, mercury, mold	n/a	n/a
22	Diet/nutrition, behavior/development, tobacco smoke, pets, lead, sun exposure, parents/teens` occupations, home injury prevention, housing age r type, firearms in the home, Indoor air pollution, water quality, heat source in the home, hobbies, molds, carbon monoxide, insecticide, pesticides, mercury, PCB`s, Asbestos, radiation exposure, nitrates, radon, Arsenic, VOC, formaldehyde, phthalates	Tobacco Smoke, lead, sun, occupations	Phthalates, formaldehyde, VOCs. Arsenic, radon
23	Tobacco Smoke	n/a	n/a
24	Tobacco Smoke	n/a	n/a
25	Tobacco Smoke	n/a	n/a
26	Indoor air pollution, environmental tobacco smoke, mold, contaminated water, smog, organic solvents, soil, lead, mercury in fish, anesthetic gases	air pollution, tobacco smoke, mold, contaminated water, smog	contaminated soil, lead, mercury
27	Tobacco Smoke	n/a	n/a
28	Radon	n/a	n/a
29	Tobacco Smoke, sun exposure, lead exposure, housing age/type, firearms in home, pets in home, parent/teen occupation, water quality, home injury prevention devices	Tobacco Smoke, sun exposure, lead exposure, housing, firearms, pets	carbon monoxide, dust mites, mold, air pollution
30	Tobacco Smoke		
31	Tobacco Smoke	n/a	n/a
32	Tobacco Smoke	n/a	n/a
33	Electromagnetic radiation	n/a	n/a
34	Tobacco Smoke	n/a	n/a
35	Tobacco Smoke	n/a	n/a
36	Lead, mold, Carbon monoxide, tobacco smoke, mercury, poor water quality, mercury, poor air quality, pesticides, PCB`s or other organic compounds	PCB's, mercury, pesticides, carbon monoxide, poor water quality,	lead, mold,
37	Tobacco Smoke, pesticides, zoonotic, molds, household exposures, wastes, radiation, water and food contamination, occupation& lifestyle, specific toxic chemicals	n/a	n/a

Table S2. *Cont.*

Author #	Exposures Assessed	Exposures Most Discussed by Clinicians	Exposures Least Discussed by Clinicians
38	Tobacco smoke	n/a	n/a
39	Tobacco Smoke	n/a	n/a
40	Tobacco smoke, animal allergens, mold exposure, insects, air pollution, indoor chemicals, dust mites	Tobacco Smoke, allergens	indoor chemical use, wood smoke, air pollution, dust mite, mold
41	Tobacco Smoke	n/a	n/a
42	Tobacco Smoke, alcohol, diet/nutrition, weight gain, job/workplace, violence, pets, seat belt use, mercury, lead, insecticides, pesticides, air pollution, VOC's, molds, PCBs, Phthalates, BPA	tobacco Smoke, alcohol, nutrition, weight gain	phthalates, BPA, PCBs, molds, VOC's
43	Behavior, diet, development, immunizations, window guards, hobbies, housing, lead, parent occupation, tobacco Smoke, heat source, asbestos, pets, carbon monoxide, radon mold, dust mites, renovation, air pollution, PCB, pesticides, VOC's, nitrates, Hg, phthalates, arsenic, sun, water, radiation	diet, air pollution, pesticides, renovation, tobacco Smoke	radon, arsenic, PCBs, mercury, phthalate, VOC's, radiation

Table S3. Proportion of clinicians responding to questionnaire items on attitude/belief, practice patterns and self-competence.

Author #	Attitude/Belief			Practice			Self-Competence	
	Environmental exposure(s) affects human health	Environmental exposure history taking should be part of regular clinical practice	Counselling of patients on Environmental exposures can help reduce exposures	Includes routine environmental exposure history in practice	Includes environmental exposure counselling in routine practice	Refers/would refer cases associated with environmental exposures to specialists	Sufficiently informed on environmental exposures	Can effectively take environmental health history
1	Most agreed	Few agreed	n/a	n/a	n/a	n/a	n/a	n/a
2	Most agreed	n/a	n/a	n/a	n/a	n/a	n/a	n/a
3	n/a	n/a	n/a	Most clinicians	n/a	n/a	n/a	n/a
4	n/a	n/a	Most agreed	Most clinicians	Most clinicians	Few clinicians	Few clinicians	n/a
5	n/a	n/a	n/a	n/a	n/a	n/a	n/a	Most clinicians
6	Most agreed	Few agreed	n/a	n/a	n/a	n/a	Few clinicians	n/a
7	Most agreed	Most agreed	Most agreed	Most clinicians	Most clinicians	n/a	Few clinicians	Few clinicians
8	n/a	n/a	n/a	n/a	n/a	n/a	Most clinicians	Most clinicians
9	n/a	n/a	n/a	Few clinicians	Most clinicians	n/a	n/a	n/a

Table S3. *Cont.*

Author #	Attitude/Belief			Practice			Self-Competence	
	Environmental exposure(s) affects human health	Environmental exposure history taking should be part of regular clinical practice	Counselling of patients on Environmental exposures can help reduce exposures	Includes routine environmental exposure history in practice	Includes environmental exposure counselling in routine practice	Refers/would refer cases associated with environmental exposures to specialists	Sufficiently informed on environmental exposures	Can effectively take environmental health history
10	Most agreed	n/a	n/a	n/a	n/a	n/a	Few clinicians	Few clinicians
11	Most agreed	n/a	Most agreed	n/a	Few clinicians	n/a	n/a	n/a
12	Few agreed	n/a	n/a	n/a	n/a	n/a	n/a	n/a
13	Most agreed	Most agreed	Few agreed	Most clinicians	n/a	n/a	n/a	n/a
14	n/a	n/a	n/a	Few clinicians	Few clinicians	n/a	n/a	n/a
15	n/a	n/a	n/a	Most clinicians	Most clinicians	n/a	Most clinicians	n/a
16	n/a	n/a	n/a	Few clinicians	n/a	n/a	Few clinicians	n/a
17	n/a	n/a	n/a	n/a	n/a	n/a	n/a	n/a
18	n/a	n/a	n/a	n/a	n/a	n/a	Few clinicians	n/a
19	n/a	n/a	n/a	Few clinicians	n/a	n/a	n/a	n/a
20	Most agreed	n/a	Most agreed	Most clinicians	n/a	n/a	n/a	n/a
21	Most agreed	Most agreed	Most agreed	Few clinicians	Few clinicians	Most clinicians	Most Clinicians	few
22	Most agreed	Most agreed	Most agreed	Few clinicians	Most clinicians	Most clinicians	Most Clinicians	n/a
23	n/a	n/a	n/a	Most clinicians	Most clinicians	Few clinicians	n/a	n/a
24	Most agreed	n/a	Most agreed	Most clinicians	Most clinicians	n/a	Few clinicians	n/a
25	n/a	n/a	n/a	n/a	n/a	n/a	Few clinicians	n/a
26	Most agreed	n/a	n/a	Most clinicians	n/a	n/a	Few clinicians	n/a
27	n/a	n/a	n/a	Most clinicians	Most clinicians	n/a	n/a	n/a
28	n/a	n/a	n/a	n/a	n/a	n/a	Few clinicians	n/a
29	Most agreed	Most agreed	n/a	Few clinicians	Few clinicians	Most clinicians	Most clinicians	n/a
30	n/a	n/a	Most agreed	Most clinicians	Most clinicians	n/a	n/a	n/a
31	n/a	n/a	Most agreed	Few clinicians	Few clinicians	n/a	n/a	n/a
32	n/a	n/a	Few agreed	n/a	n/a	n/a	n/a	n/a

Table S3. *Cont.*

Author #	Attitude/Belief			Practice			Self-Competence	
	Environmental exposure(s) affects human health	Environmental exposure history taking should be part of regular clinical practice	Counselling of patients on Environmental exposures can help reduce exposures	Includes routine environmental exposure history in practice	Includes environmental exposure counselling in routine practice	Refers/would refer cases associated with environmental exposures to specialists	Sufficiently informed on environmental exposures	Can effectively take environmental health history
33	Most agreed	n/a	n/a	n/a	n/a	n/a	n/a	n/a
34	n/a	n/a	n/a	Most clinicians	Most clinicians	n/a	Few clinicians	n/a
35	Most agreed	n/a	Most agreed	Most clinicians	Most clinicians	n/a	n/a	n/a
36	Most agreed	Most agreed	n/a	n/a	n/a	Most clinicians	Most clinicians	n/a
37	n/a	Most agreed	Most agreed	Few clinicians	n/a	n/a	Most clinicians	n/a
38	Most agreed	n/a	n/a	n/a	Few clinicians	n/a	n/a	n/a
39	n/a	n/a	n/a	n/a	Most clinicians	Few clinicians	n/a	n/a
40	n/a	n/a	n/a	n/a	Most clinicians	n/a	Few clinicians	n/a
41	n/a	n/a	n/a	Few clinicians	Few clinicians	n/a	n/a	n/a
42	Most agreed	Most agreed	Most agreed	Most clinicians	Most clinicians	n/a	Most clinicians	n/a
43	Most agreed	Most agreed	Few agreed	n/a	n/a	Most clinicians	Few clinicians	

Note: * n/a—Not Applicable.

Table S4. Showing proportion of clinicians responding to questionnaire items on clinician training and obstacles to practice.

Author #	Training			Support and Follow-up		Obstacles to Practice
	Prior training in environmental health history taking	Requires/interested in more training	Most helpful source for continuing environmental health education	Practitioners role in changing social, psychological and physiological environments of patients	Receives/requests feedback from patients	
1	n/a	n/a	n/a	n/a	n/a	n/a
2	n/a	Most clinicians	n/a	n/a	n/a	n/a
3	n/a	n/a	n/a	n/a	n/a	n/a
4	n/a	n/a	n/a	n/a	n/a	time, practice efficiency, patients response
5	n/a	n/a	n/a	n/a	n/a	Training, environmental data, Eh consultation services, laboratory support, financial incentive

Table S4. *Cont.*

Author #	Training			Support and Follow-up		Obstacles to Practice
	Prior training in environmental health history taking	Requires/interested in more training	Most helpful source for continuing environmental health education	Practitioners role in changing social, psychological and physiological environments of patients	Receives/requests feedback from patients	
6	Few clinicians	Most clinicians	Environmental educational programs, internet; colleagues	n/a	n/a	training, time
7	Few clinicians	n/a	Agencies/associations, newsletter,	n/a	n/a	n/a
8	n/a	n/a	n/a	n/a	n/a	training
9	n/a	n/a	n/a	n/a	n/a	patients response, training, language, time, reimbursement
10	Few clinicians	n/a	n/a	n/a	n/a	Training, lack of knowledge, poor coordination
11	n/a	Most clinicians	n/a	n/a	n/a	Time, patients response, lack of educational resource, lack of adequate reimbursement, lack of training
12	n/a	n/a	n/a	n/a	n/a	n/a
13	Few clinicians	Most clinicians	guidelines	n/a	n/a	n/a
14	n/a	n/a	n/a	n/a	n/a	n/a
15	n/a	n/a	n/a	n/a	n/a	patients response, time
16	Most clinicians	Most clinicians	texts; internet; colleague experts	n/a	n/a	n/a
17	n/a	Most clinicians	n/a	n/a	n/a	resources, training
18	n/a	n/a	n/a	n/a	n/a	n/a
19	Few clinicians	Few clinicians	agencies, internet, colleagues	n/a	n/a	n/a
20	n/a	Most clinicians	journals, continuing education	n/a	n/a	Time, patients concern, training, lack of appropriate reimbursement
21	Few clinicians	Most clinicians	n/a	n/a	n/a	time, resources
22	Few clinicians	Most clinicians	n/a	Most agreed	n/a	n/a
23	Few clinicians	Most clinicians	n/a	n/a	Few clinicians	patients response, training, competing priorities, resources, time
24	n/a	n/a	n/a	n/a	Few clinicians	training, time, resources
25	n/a	n/a	n/a	n/a	n/a	n/a

Table S4. *Cont.*

Author #	Training			Support and Follow-Up		Obstacles to Practice
	Prior training in environmental health history taking	Requires/interested in more training	Most helpful source for continuing environmental health education	Practitioners role in changing social, psychological and physiological environments of patients	Receives/requests feedback from patients	
26	Few clinicians	n/a	journals, internet, workshops, conferences	n/a	n/a	n/a
27	Few clinicians	n/a	n/a	n/a	n/a	n/a
28	n/a	n/a	n/a	n/a	n/a	n/a
29	Few clinicians	Most clinicians	journals, lectures, agencies, presentations	n/a	n/a	resources (referral centers)
30	Most clinicians	Most clinicians	n/a	n/a	n/a	time, reaching fathers and socially vulnerable families
31	n/a	Most clinicians	n/a	n/a	n/a	patients response, resources, training
32	Most clinicians	n/a	n/a	n/a	n/a	n/a
33	Few clinicians	n/a	n/a	n/a	n/a	n/a
34	Few clinicians	n/a	n/a	n/a	n/a	n/a
35	Few clinicians	Most clinicians	n/a	n/a	n/a	time, interest, patients response,
36	Few clinicians	Most clinicians	n/a	n/a	n/a	resources(referral centers
37	n/a	n/a	Textbooks, guideline; internet, conferences, journals	n/a	n/a	time, training
38	Few clinicians	n/a	n/a	n/a	n/a	n/a
39	n/a	n/a	n/a	n/a	n/a	n/a
40	n/a	n/a	n/a	n/a	n/a	n/a

Table S4. *Cont.*

Author #	Training			Support and Follow-up		Obstacles to Practice
	Prior training in environmental health history taking	Requires/interested in more training	Most helpful source for continuing environmental health education	Practitioners role in changing social, psychological and physiological environments of patients	Receives/requests feedback from patients	
41	n/a	n/a	n/a	n/a	n/a	Training, patients concern
42	Few clinicians	n/a	Agencies, Internet	Few agreed	n/a	Lack of knowledge, patients concern, complexity of topic
43	Few clinicians	n/a	n/a	n/a	n/a	n/a

Note: * n/a—Not Applicable.

© 2015 by the authors; licensee MDPI, Basel, Switzerland. This article is an open access article distributed under the terms and conditions of the Creative Commons Attribution license (http://creativecommons.org/licenses/by/4.0/).
